# Effects of NaCl Treatment on Flavonoid Biosynthesis and Antioxidant System During Buckwheat Germination

**DOI:** 10.3390/plants15060904

**Published:** 2026-03-14

**Authors:** Miaoyao Yu, Meixia Hu, Dehcontee Diana Adams, Meilin Wang, Zhengfei Yang, Jiangyu Zhu, Yongqi Yin

**Affiliations:** College of Food Science and Engineering, Yangzhou University, Yangzhou 225009, China; mz120242252@stu.yzu.edu.cn (M.Y.); mz120222068@stu.yzu.edu.cn (M.H.); mh25213@stu.yzu.edu.cn (D.D.A.); mx320231388@stu.yzu.edu.cn (M.W.); yzf@yzu.edu.cn (Z.Y.); 008051@yzu.edu.cn (J.Z.)

**Keywords:** phenylpropane metabolism, functional foods, NaCl treatment, flavonoid compounds

## Abstract

Exposure to abiotic stresses commonly stimulates the production of secondary metabolites in plants, and flavonoids represent a major class of these bioactive compounds. NaCl effects on antioxidant system treatment and flavonoid production in buckwheat sprouts was examined in this study using buckwheat as the raw material. In order to clarify the regulatory function of NaCl in these physiological processes, the changes in pertinent indices of buckwheat sprouts exposed to the control and NaCl treatments were studied. The results indicated that at three days old, the sprouts subjected to 80 mM NaCl treatment exhibited the highest total flavonoid content. The significant increase in enzyme activity (cinnamate 4-hydroxylase and 4-coumaroyl-CoA ligase, etc.) responsible for flavonoid biosynthesis provides strong evidence for this conclusion. The antioxidant system in buckwheat was activated by NaCl treatment, as evidenced by the dramatically increased antioxidant enzyme activities and the relative levels of expression of their respective genes compared to the control group. Levels of malondialdehyde and hydrogen peroxide were markedly higher than those in the control group, indicating that NaCl treatment inhibited the growth of buckwheat sprouts. This study not only reveals the mechanisms underlying buckwheat’s response to NaCl stress but also lays a theoretical foundation for developing functional foods enriched with flavonoid-rich buckwheat sprouts.

## 1. Introduction

As an important group of plant secondary metabolites, flavonoids function as key protective compounds that mediate plant responses to biotic and abiotic stresses. [1]. Salt stress, as a common abiotic stressor, has recently been acknowledged for its role in enhancing the accumulation of bioactive compounds in plants. Relevant research has demonstrated that NaCl application effectively stimulates the biosynthesis of secondary metabolites, such as flavonoids and phenolic compounds, during seed germination in crops, including soybean [2], maize [3], and millet [4]. Flavonoids exhibit multiple pharmacological activities, including antihypertensive, anticancer, antioxidant, and antiviral effects [5,6]. Research indicates that paclitaxel, a flavonoid derivative found in natural plants, inhibits tumor growth by modulating the signaling pathways [7]. Additionally, dauricine interferes with specific cyclins and activates caspases, thereby impeding tumor cell proliferation and promoting apoptosis [8,9]. Flavonoids can effectively prevent insulin resistance and oxidative stress initiated by high-fructose diets by reducing insulin concentrations and enhancing insulin signaling molecules [10]. Buckwheat, as an emerging functional food ingredient, is rich in flavonoid bioactive compounds [11], with numerous processing applications within the food, health supplement, and pharmaceutical sectors [12]. Due to the high flavonoid content in buckwheat and the close relationship between flavonoids and human health requirements, buckwheat has emerged as an essential raw ingredient in functional food formulation and phytopharmaceutical products.

To increase flavonoid levels in Fagopyrum esculentum, our previous findings indicate that germination enhances both the enzymatic activities and transcriptional expression of cinnamate 4-hydroxylase (C4H), chalcone isomerase (CHI), 4-coumaroyl-CoA ligase (4CL) and phenylalanine ammonia-lyase (PAL) in the flavonoid biosynthetic pathway, thereby facilitating flavonoid production and accumulation. [13]. The biosynthesis of flavonoids in plants originates from the common phenylpropanoid pathway and is subsequently synthesized through branch pathways of phenylpropanoid metabolism [14] and proceeds through branch-specific enzymatic reactions involving a series of complex enzyme-mediated steps, ultimately leading to the formation of flavonoid compounds [15]. Flavonoid accumulation in plants is associated with various factors; upon experiencing abiotic stress, plants activate their antioxidant systems, leading to flavonoid enrichment. Under UVB and extreme temperature stress, UVB irradiation enhances flavonoid accumulation through the upregulation of gene expression and increased activities of key enzymes involved in flavonoid biosynthesis, including chalcone synthase and phenylalanine ammonia-lyase, thereby enhancing flavonoid biosynthesis in soybean [16]. Tomato seedlings subjected to heat shock treatment exhibit a significant increase in flavonoid content, thereby enhancing their antioxidant capacity [17].

Under 60 mM NaCl exposure, pea sprouts displayed higher total phenolic levels, along with enhanced activities of PAL, C4H, and 4CL, thereby augmenting the antioxidant capacity of the sprouts [18]. Moreover, NaCl application stimulates essential enzyme activity and enhances the levels of transcriptionally associated genes, thereby promoting the biosynthesis of secondary metabolites. Relevant studies indicate that 150 mM NaCl stress enhances the biosynthesis and accumulation of resveratrol by upregulating 4CL activity and C4H, PAL, and 4CL relative levels of expression in the cotyledons, while concurrently increasing PAL and C4H activities and PAL and 4CL relative expression levels in non-cotyledonous tissues [19]. Significant upregulation of CHS (chalcone synthase) and CHI expression levels in mung bean sprouts was observed following treatment with 50 mM NaCl [20]. NaCl treatment can modulate transcription factors to enhance plant tolerance to stress. Previous studies have shown that NaCl stress significantly increases the relative transcription levels of the *FtNHX1* and *FtSOS1* genes. [21]. The aforementioned studies indicate that NaCl treatment promotes the enrichment of functional components in plants; however, they do not investigate or analyze the regulatory mechanisms of the antioxidant system in buckwheat under NaCl stress or the biosynthesis of flavonoid compounds. Our study not only investigates the antioxidant system regulatory mechanisms and the biosynthesis of flavonoid compounds under NaCl stress but also includes an analysis of the underlying molecular mechanisms.

Based on our previous research [13], this study selected “Pin Tian 2” buckwheat seeds as the material. Under NaCl treatment conditions, we examined how NaCl regulates flavonoid biosynthesis in buckwheat sprouts and explored the allied molecular mechanisms. This study also offers a theoretical basis for the processing of flavonoid-rich buckwheat sprouts as a raw food material.

## 2. Results

### 2.1. Growth Status, MDA Content, and H_2_O_2_ Levels

**Figure 1:**
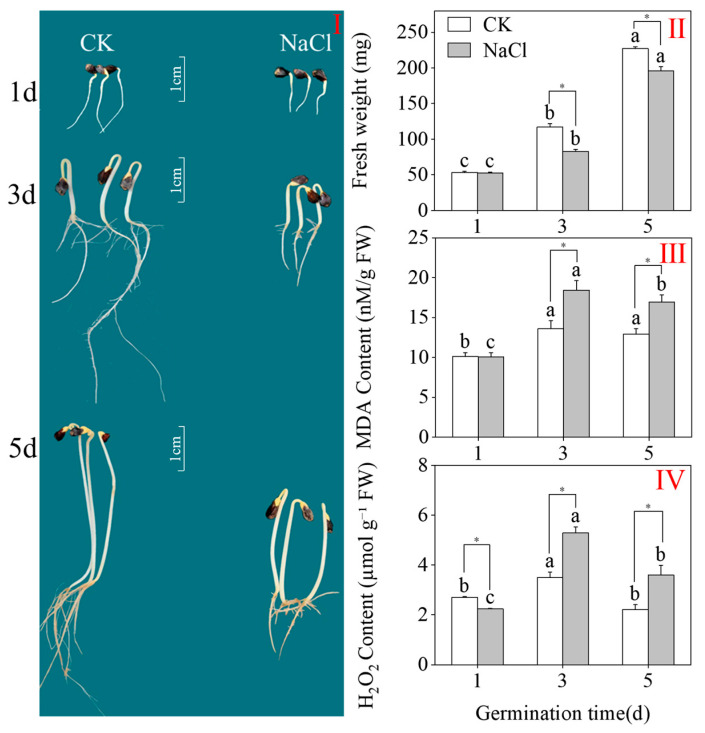
The effects of NaCl treatment on growth status (**I**), fresh weight (**II**), MDA levels (**III**), and H_2_O_2_ levels (**IV**) in buckwheat shoots. Unique lowercase letters denote statistically important variations among treatments within the same group (one-way ANOVA with Tukey’s test; *p* < 0.05). * denotes a significant difference between the CK and NaCl results at the same germination time (Student’s *t*-test, *p* < 0.05).

As shown in Figure 1I, NaCl significantly inhibited seedling growth in three-day-old sprouts. As Figure 1II demonstrates, in comparison to the CK, the fresh weight of three-day-old sprouts decreased by 29% under NaCl treatment (*p* < 0.05; one-way ANOVA), whereas the fresh weight of five-day-old sprouts decreased by 13.6%.

The levels of MDA and H_2_O_2_ serve as indicators of the degree of stress experienced by the plants. With prolonged germination, the contents of MDA and H_2_O_2_ in the sprouts initially increased and then declined (Figure 1III,IV). Compared to the CK, NaCl treatment significantly elevated the MDA and H_2_O_2_ levels in buckwheat sprouts at both three and five days. Under NaCl stress, the MDA and H_2_O_2_ contents in three-day-old sprouts increased by 35.29% and 52.94%, respectively, reaching their peak values (18.44 nM/g FW and 5.29 mM/g FW, respectively). NaCl stress led to a significant increase in MDA and H_2_O_2_ contents in three-day-old sprouts, with both reaching their peak levels during the early stages of stress. These findings suggest that NaCl treatment markedly elevates MDA and H_2_O_2_ contents in three-day-old sprouts.

### 2.2. Total Phenolic Content and Total Flavonoid Content

**Figure 2:**
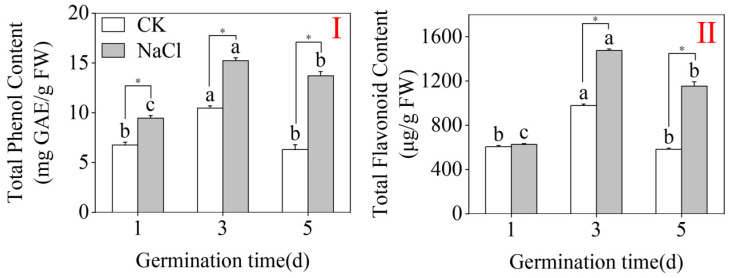
The effects of NaCl treatment on total phenolic (**I**) and total flavonoid (**II**) contents in buckwheat buds. Distinct lowercase letters denote important variations among treatments within the same group (one-way ANOVA with Tukey’s post hoc test; *p* < 0.05). * denotes a significant difference between the CK and NaCl results at the same germination time (Student’s *t*-test; *p* < 0.05).

Figure 2 demonstrates that, under the CK condition, total phenolic and flavonoid levels peaked in three-day-old buckwheat buds at 10.48 mg GAE/g FW and 979.35 µg/g FW, correspondingly (*p* < 0.05; one-way ANOVA). Compared to CK, NaCl treatment significantly elevated the flavonoid and phenolic levels in both three- and five-day-old sprouts (Figure 2I). Specifically, NaCl application resulted in peak concentrations of 15.23 mg GAE/g FW for total phenolics and 1476.54 µg/g FW for total flavonoids in three-day-old sprouts. The flavonoid content was highly amplified, which is presumed to be an adaptive strategy of buckwheat sprouts to cope with salt stress. These findings suggest that NaCl treatment substantially promotes biosynthesis and phenolic compound accumulation as well as flavonoid concentration in buckwheat buds.

### 2.3. Antioxidant Capacity

**Figure 3:**
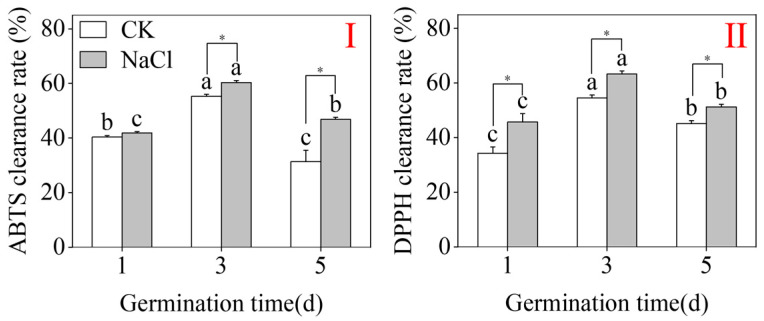
The effects of NaCl treatment on ABTS (**I**) and DPPH (**II**) radical scavenging activities in buckwheat sprouts. Unique lowercase letters within the same parameter show statistically significant variations among treatments within the same group (one-way ANOVA followed by Tukey’s test; *p* < 0.05). * denotes a significant difference between CK and NaCl results at the same germination time (Student’s *t*-test, *p* < 0.05).

As shown in Figure 3, the CK treatments reached their peak values at three days. Compared to CK, NaCl treatment highly improved the DPPH scavenging activity in three-day-old spouts (Figure 3II) (*p* < 0.05; one-way ANOVA). Under NaCl treatment, ABTS and DPPH scavenging activities in three-day-old buds were 60.26% and 63.28%, respectively. These results indicate that both CK and NaCl treatments achieved maximum rates of ABTS and DPPH scavenging at three days, with NaCl treatment further improving the antioxidant capacity of the shoots, meaning that exogenous stress successfully activated the antioxidant system in buckwheat sprouts.

### 2.4. Relative Activity of Antioxidant Enzymes and the Corresponding Gene Expression Levels

**Figure 4:**
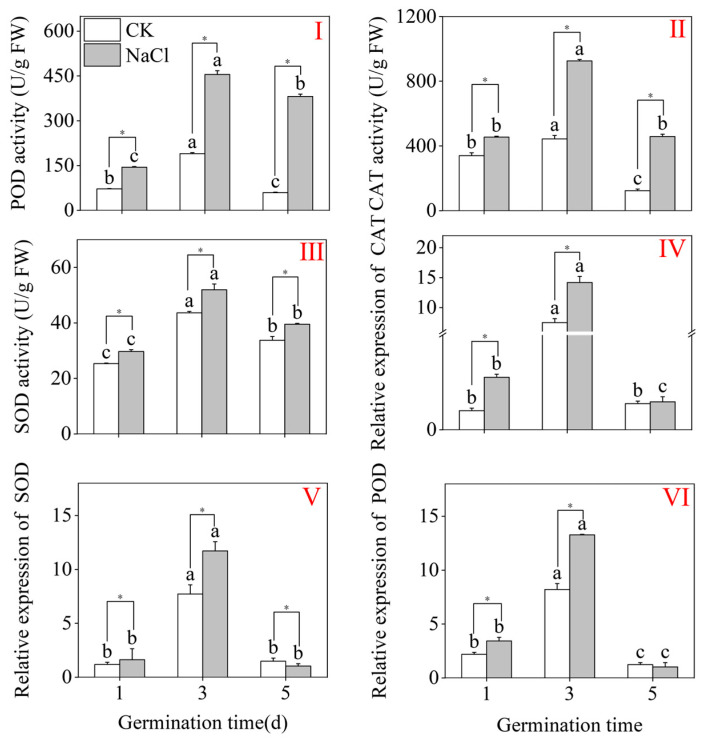
The effects of NaCl treatment on the CAT (**II**), POD (**I**), and SOD (**III**) activities in buckwheat sprout seedlings, and the relative levels of CAT (**IV**), SOD (**V**), and POD (**VI**) expression. Varied lowercase letters within the same parameter show statistically significant variations among treatments within the same group (one-way ANOVA with Tukey’s test; *p* < 0.05). * denotes a significant difference between the CK and NaCl results at the same germination time (Student’s *t*-test; *p* < 0.05). U/g FW: Units per gram fresh weight. Gene expression was quantified using the 2^−ΔΔCt^.

Figure 4 indicates that the highest value under CK treatment was observed on day 3. Compared to CK, NaCl treatment markedly enhanced the enzymatic activities of POD, CAT, and SOD in three-day-old buds (Figure 4I–III). Under NaCl treatment, CAT, POD, and SOD activities peaked at three days, reaching 454.98 U/g FW, 925.78 U/g FW, and 51.95 U/g FW (*p* < 0.05; one-way ANOVA), respectively. These values represent 1.40-fold, 1.08-fold, and 1.18-fold increases when compared to the CK, respectively. Concurrently, the POD, SOD, and CAT expression levels in three-day-old buckwheat shoots were markedly enlarged (Figure 4IV–VI). Relative to the CK, NaCl treatment markedly enhanced the expression of CAT, POD, and SOD in three-day-old buckwheat sprouts by 1.88-fold, 3.30-fold, and 5.7-fold, correspondingly (Figure 4IV–VI). These outcomes indicate that NaCl treatment meaningfully reinforces the antioxidant system in buds by increasing both antioxidant enzyme activities and the comparative levels of expression of related genes; notably, the expression levels of related genes may indicate a consistent upregulation trend with enzyme activity.

### 2.5. Relative Expression Levels of Key Flavonoid Biosynthetic Enzyme Activities and Their Corresponding Genes

**Figure 5:**
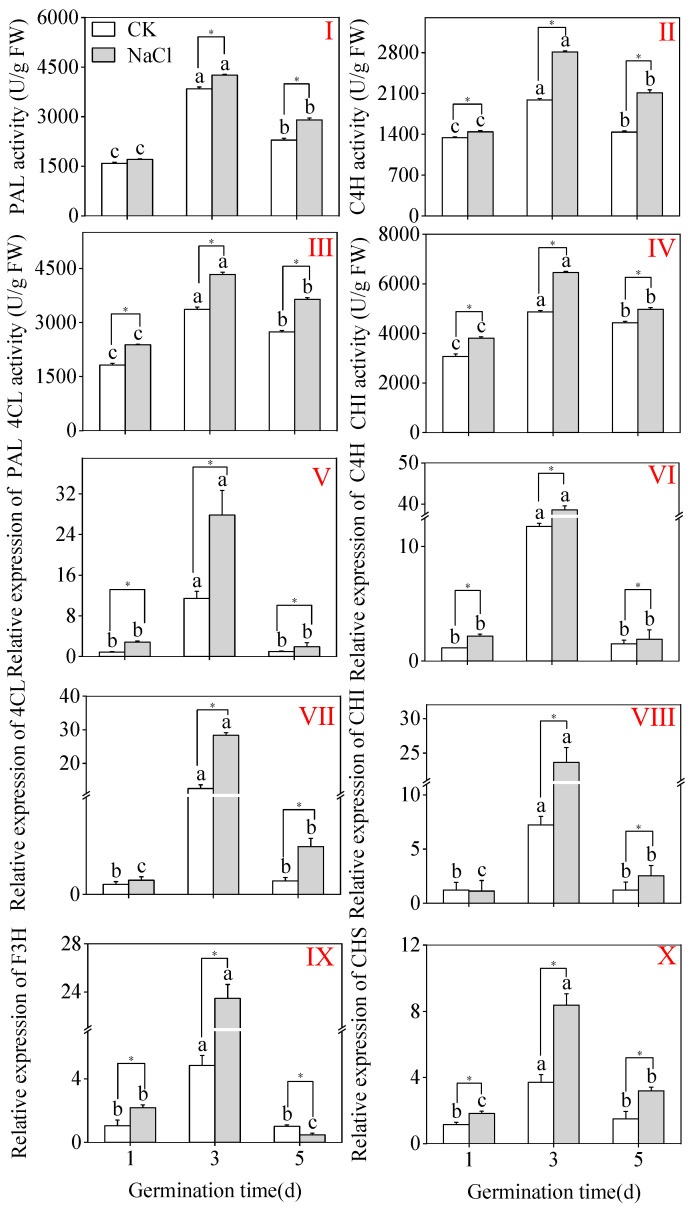
The effects of NaCl treatment on the activities of crucial flavonoid biosynthetic enzymes C4H (**II**), PAL (**I**), 4CL (**III**), and CHI (**IV**), as well as the relative gene levels of expression of C4H (**VI**), PAL (**V**), CHI (**VIII**), 4CL (**VII**), F3H (**IX**), and CHS (**X**) in buckwheat buds. Different lowercase letters within the same parameter indicate statistically significant variations among same group treatments (one-way ANOVA with Tukey’s test; *p* < 0.05). * denotes a significant difference between the CK and NaCl results at the same germination time (Student’s *t*-test; *p* < 0.05). U/g FW: Units per gram fresh weight. Gene expression was enumerated using the relative 2^−ΔΔCt^.

As shown in Figure 5, the CK treatments reached their peak values at three days. Compared to the CK, the treatment of NaCl majorly increased the enzymatic C4H, PAL, 4CL, and CHI activities in three-day-old shoots (Figure 5I–IV) (*p* < 0.05; one-way ANOVA). Under NaCl treatment, the activities of PAL, C4H, 4CL, and CHI peaked at three days, reaching 4255.26 U/g FW, 2188.97 U/g FW, 4334.29 U/g FW, and 6454.33 U/g FW, respectively, which are evidently higher than those observed in the CK group. Additionally, the expression levels of genes related to flavonoid biosynthesis enzymes in three-day-old sprouts under NaCl treatment were significantly higher than at other germination stages (Figure 5V–X). Compared to the CK, NaCl treatment notably enhanced the expression levels of C4H, PAL, CHI, 4CL, and F3H in three-day-old shoots (Figure 5V–X). These results specify that NaCl treatment can enhance the action of key metabolic enzymes involved in flavonoid biosynthesis in buckwheat buds significantly. The upregulation amplitude of some gene expressions was higher than the increase in enzyme activity. It is speculated that there may be post-translational regulation, which requires further research for verification.

### 2.6. The Effect of NaCl Treatment on the Relative Expression Levels of Stress-Responsive Genetic Factor in Buckwheat Shoots

**Figure 6:**
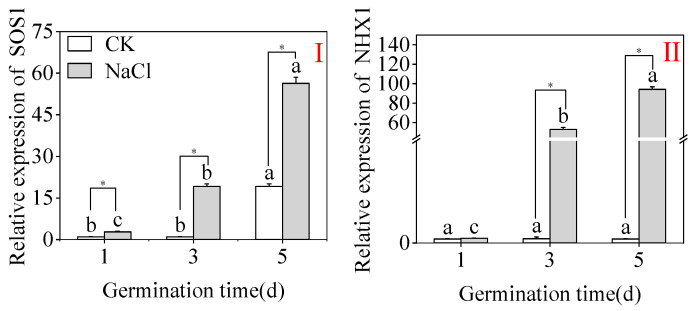
The effects of NaCl treatment on the comparative expression levels of stress-related genes in buckwheat buds, specifically SOS1 (**I**) and NHX1 (**II**). Different lowercase letters within the same parameter show statistically important changes among treatments within the group. * denotes a significant difference between the CK and NaCl results at the same germination time (Student’s *t*-test; *p* < 0.05). Gene expression was measured using the relative 2^−ΔΔCt^.

As demonstrated in Figure 6, compared to the control (CK), NaCl treatment meaningfully amplified the expression levels of stress-related genes, with the relative expression of *SOS1* and *NHX1* continuously upregulated during germination. On day 5, the relative expression of *SOS1* in the NaCl-treated group was 19 times higher than that in the CK group, while the relative expression of *NHX1* was 49.45 times higher than that in the CK group. The results obtained show that continuous upregulation of *SOS1* and *NHX1* gene expression suggests that buckwheat sprouts adapt to salt stress through molecular regulation, although the direct link between this mechanism and flavonoid biosynthesis requires further validation.

## 3. Discussion

Numerous studies have demonstrated that NaCl treatment promotes the biosynthesis of secondary metabolites, such as flavonoids and phenolic compounds, during the germination of plants including soybean [2], maize [3], and millet [4]. This was ascribed mostly to the fact that NaCl stimulated the plant’s antioxidant defense system, in which flavonoids, as essential antioxidant molecules, played a vital part, scavenging reactive oxygen species (ROS) and defending cells against oxidative impairment, thus enhancing their biosynthesis.

Plants’ phenylpropanoid pathway is regarded as one of the main pathways towards the accumulation of flavonoid secondary metabolites, and the activity level of the gene expression of major enzymes determines the combination and accumulation of the flavonoid secondary metabolites. Research has demonstrated that the activities of alpha-galactosidase and the total flavonoid content, CHI and PAL in buckwheat sprouts are positively correlated [22]. This research showed that the activities of CHI, C4L, PAL, and C4H in buckwheat buds were significantly enhanced by NaCl treatment in a comparison with non-treated samples. This improvement indicates that NaCl may stimulate the buildup of total flavonoids by adjusting the phenylpropanoid metabolic pathway. These results are in line with past reports which suggest that electromagnetic fields and weakly acidic treatments with electrolyzed water can cause alterations in the phenylpropanoid pathway in buckwheat [23]. This study focused on the Pin Tian 2 cultivar, examining germination under varying NaCl concentrations and revealing significant differences in total flavonoid accumulation. Previous studies have demonstrated that a NaCl concentration of 60 mM significantly enriches flavonoid compounds in soybeans [24], further confirming that different cultivars exhibit varying responses to NaCl stress. During germination under salt stress, flavonoid accumulation also varied among the buckwheat varieties. Initial investigations have revealed that buckwheat sprouts attain their highest flavonoid content at 10 days [25], 7 days [26] and 6 days [27], respectively. Our results have shown that the total flavonoid content tends to increase and then decrease, reaching its highest point at 3 germination days. These differences can be explained by the fact that the cultivars and germination conditions were different.

NaCl treatment in the present study had a significant effect on the activities of C4H, CHI, C4L, and PAL in buckwheat buds in comparison to the control samples. This amplification shows that NaCl enhances the amassment of total flavonoids by regulating the phenylpropanoid metabolic pathway. Besides enzyme action, the comparative expression levels of six main genes were also examined. NaCl treatment improved the expression of *FeF3H*, *FeC4H*, *FeCHS*, *Fe4CL*, *FeCHI*, and *FePAL* over three days of germination significantly. Other reports have also indicated that the expression of key genes in buckwheat sprouts is also significantly induced by germination [28], microwave exposure [29], and phenylalanine treatment [30]. It was discovered that microwave-treated buckwheat sprouts have different abundances of proteins involved in flavonoid biosynthesis and the phenylpropanoid pathway. Additional studies in the future involving transcription factor regulation as well as the use of new omics technologies like proteomics, metabolomics, and transcriptomics can give extra information on the processes that control flavonoid enrichment in buckwheat. In this study, it was noted that NaCl treatment suppressed buckwheat bud growth and caused a significant increase in MDA and H_2_O_2_, which showed that the accumulation of ROS and cell membrane destruction caused by lipid peroxidation occurred. Exposure to NaCl leads to the formation of osmotic stress and the removal of water in the cytoplasm, leading to cellular dehydration and subsequent loss of both vacuole and cytoplasmic volume. But over an extended period of evolution, buckwheat has acquired a strong self-defense system to overcome endogenous free radicals. The antioxidant defense system is also correspondingly adapted to endure under NaCl-induced stress as the levels of bioactive compounds like flavonoids and phenolics change. The findings show that NaCl treatment enormously improved the activity and expression of the major antioxidant enzymes of buckwheat sprouts such as SOD, POD, and CAT compared to normal germination conditions. This was likely due to the NaCl treatment activating the antioxidant system in buckwheat sprouts, as evidenced by a significant increase in both the activity of related antioxidant enzymes and their corresponding gene expression levels compared to the control group. The joint action of the elevated gene expression and enzyme activity of antioxidant enzymes helps the removal of reactive oxygen species, which subsequently leads to the protection of the plant cells against degrading effects. This observation is in line with earlier reports that indicated that soybean [31,32], buckwheat [33], wheat [34,35], and *Elymus sibiricus* [36] react to changes in the environment by improving the process of antioxidant system activation. This may indicate that NaCl treatment has a significant effect on enhancing the antioxidant ability of buckwheat sprouts including ABTS and DPPH radical scavenging activity. In the same manner, previous researchers have indicated that microwave irradiation [37,38], laser treatment [39] and trace element water treatment [40] can be used to increase the antioxidant capacity of buckwheat buds.

In conclusion, NaCl treatment produced the maximum quantity of total flavonoids and overall phenolics in buckwheat sprouts following three days of germination. NaCl treatment significantly enhanced the activities of key enzymes involved in the phenylpropanoid pathway, as well as the expression of associated genes. Also, NaCl treatment elevated the functions of antioxidant enzymes and their respective gene expressions in buckwheat buds. But NaCl treatment restrained the growth and development of buckwheat sprouts as indicated by the significant changes in the appearance of stress-associated genes and MDA and H_2_O_2_ levels (*p* < 0.05).

## 4. Materials and Methods

### 4.1. Germination Treatment and Experimental Design

Buckwheat seeds: The variety was Pin Tian 2, which was offered by the Crop Genetic Resources Institute of Jiangsu Academy of Agricultural Sciences in 2022. The preservation of the seeds was done at −18 C. Before the experiment, buckwheat seeds were disinfected in a 1% solution of sodium hypochlorite for 15 min, then cleaned with purified water until the pH became neutral. The seeds were then moistened in distilled water in darkness at 30 °C using a germination chamber. One hundred surface-sterilized seeds were placed in each germination tray (dimensions: 15 cm × 10 cm × 5 cm), with sterile filter paper (3 layers) used as the germination substrate. Distilled water and NaCl solution were applied via spray simultaneously every 12 h. The CK and NaCl seeds were simultaneously sprayed with 30 mL. Samples were randomly collected at 1, 3, and 5 days of germination and kept at −20 °C for future examination. Different seed treatments were applied: (1) CK, control, with distilled water spray; (2) NaCl stress treatment at 80 mM concentration. The NaCl concentration was selected based on preliminary screening (Supplementary Figure S1). (The NaCl reagent used in this experiment was purchased from Sinopharm Chemical Reagent Co., Ltd. (Shanghai, China) with a purity of ≥99.5%. The main impurities include trace sulfate (≤0.02%), chloride (≤0.005%), and heavy metals (≤0.001%). Detailed specifications are available in the product manual. A Millipore ultrapure water system was used for both the preparation of the NaCl solution and the experiments.)

A random sample of 30 buckwheat sprouts was selected, and the length of each sprout was measured using a vernier caliper with an accuracy of 0.01 cm, while the fresh weight was determined using an analytical balance with a precision of 0.001 g. Each treatment was conducted with three biological replicates, and 2 g of seedlings was randomly selected for each replicate. All measurements were performed with three technical replicates

### 4.2. Physiological and Biochemical Indicators

The malondialdehyde (MDA) determination was carried out based on the procedure reported by Zhuang [41]. A certain quantity of the sample was drawn and combined with trichloroacetic acid. The supernatant was removed and kept in the dark. A portion of the supernatant was, in turn, mixed with the thiobarbituric acid solution. The mixture was centrifuged once more after a quick cooldown and the reaction solution was harvested. OD_600 nm_, OD_532 nm_ and OD_450 nm_ were measured.

The determination of hydrogen peroxide (H_2_O_2_) was performed following the method described by Xue [42]. A measured amount of sample was homogenized with buffer solution. The mixture was centrifugated while the supernatant was collected. An aliquot of the supernatant was then added to the reaction solution, and absorbance was determined at OD_390nm_ and OD_593nm_.

### 4.3. Total Flavonoids and Total Phenolics

The total flavonoid content was estimated by using the procedure outlined by Yin et al. [43]. A given portion of the sample was mixed with ethanol, then the mixture was ultrasonicated and centrifuged. The supernatant was removed and diluted and OD_260nm_ absorbance was measured.

The phenolic content was enumerated using the process outlined by Mencin et al. [44]. One of the samples was centrifuged and a measured volume of the sample was mixed in methanol and subsequently centrifuged. A supernatant was prepared by taking an aliquot of the resultant and adding Folin–Ciocalteu reagent as well as Na_2_CO_3_ and letting the reaction proceed in the dark. OD_765nm_ absorbance was then measured.

### 4.4. Antioxidant Capacity

The determination of 1,1-Diphenyl-2trinitrophenylhydrazine (DPPH) and 2,2′-azino-bis(3-ethylbenzothiazoline-6-sulfonic acid) (ABTS) radical scavenging activities was conducted following the methodology described by Zhang et al. [45].

### 4.5. Activity of Antioxidant Enzymes

Peroxidase (POD), catalase (CAT) and superoxide dismutase (SOD) activities were assessed following the method highlighted by Huang et al. [46]. A defined quantity of the sample was homogenized in sodium phosphate buffer, and the resulting supernatant was collected for subsequent enzymatic analysis. One unit of SOD and APX activity is defined as the amount of enzyme required to cause a change of 0.01 per minute in absorbance at OD_560nm_ and OD_290nm_, respectively. Similarly, one unit of CAT and POD activity is defined as the quantity of enzyme resulting in a change of 0.01 per minute in absorbance at OD_240nm_ and OD_470nm_, respectively.

### 4.6. Activity of Flavonoid Metabolism-Related Enzymes

The activity of CHI, 4CL, C4H and PAL was assessed as described by Yin et al. [24]. A certain portion of the sample was homogenized in Tris–HCl buffer and then centrifuged to separate the supernatant. A unit represented a 0.01 modification in absorbance per minute at OD_290nm_, OD_340nm_, OD_333nm_, or OD_381nm_.

### 4.7. Key Gene Expression Levels

#### 4.7.1. RNA Extraction and Reverse Transcription

After grinding the buckwheat into powder using liquid nitrogen and transferring it to a centrifuge tube, add lysis buffer rapidly and mix thoroughly. Once lysis is complete, extract RNA using the RNA extraction kit (R6827, Omega, Norcross, GA, USA). Perform reverse transcription with the RNA reverse transcription kit (RR047A, TAKARA, Kyoto, Japan), then store the resulting cDNA at −80 °C for future use.

#### 4.7.2. qPCR Primers

Table 1 presents the primer sequences for the target genes and the internal reference gene (Actin), based on the complete genome sequence of buckwheat published in the NCBI database and the gene primers reported in the relevant literature [21,47,48].

#### 4.7.3. qPCR Reaction Conditions and Establishment of the Quantitative PCR System

Quantitative PCR was performed using TAKARA’s TB Green Premix Dimer Eraser (RR091A), TAKARA, Japan, Kyoto. The expression level of the reference gene (Actin) was normalized to 1. Genetic expression was measured using the 2^−ΔΔCt^ [43]. Based on the instructions for the TB Green Premix Dimer Eraser (RR091A, TAKARA, Japan), the specific parameters for the quantitative PCR reaction system and the PCR amplification program were established. The annealing temperature was 72 °C. Amplification efficiency ranged from 95% to 104%.

### 4.8. Processing of Data and Statistical Analysis

The results from the experiment are presented as mean ($\bar{X}$) ± standard deviation (SD), based on data from three autonomous tests. Statistical analysis was achieved using one-way analysis of variance (ANOVA), followed by Tukey’s multiple comparison test to assess differences between group means. Statistical relevance was assessed at the 0.05 level (*p* < 0.05). All data were scrutinized using DPS software (Version 9.0, China), and data visualization was conducted with Origin 2022.

## 5. Conclusions

**Figure 7:**
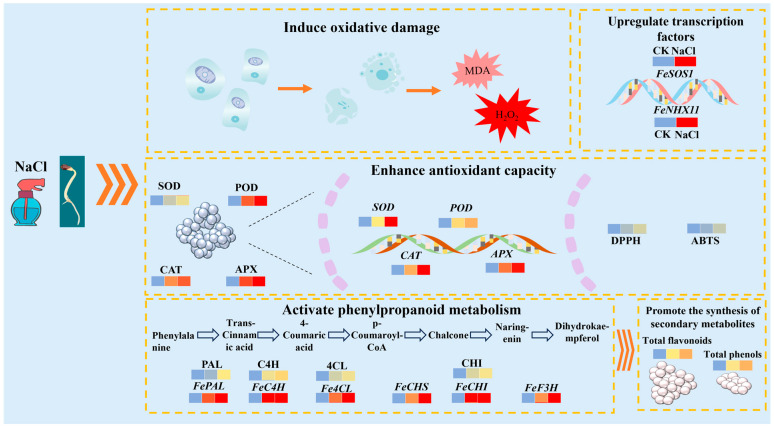
A hypothetical model for the regulatory effects of NaCl treatment on the physiological metabolism and flavonoid biosynthesis in buckwheat sprouts.

This study found that both total flavonoid and phenolic contents in buckwheat buds reached their highest levels after 3 days of NaCl treatment. The NaCl-treated sprouts exhibited enhanced activities of important enzymes in the phenylpropanoid metabolic pathway and upregulated expression of related genes. Additionally, NaCl treatment increased antioxidant enzyme activity and the expression of their corresponding genes in buckwheat shoots. This study identified a germination treatment of 80 mM NaCl for 3 days, which enhanced the flavonoid content and antioxidant activity of buckwheat shoots, thereby offering a theoretical basis for the production of functional buckwheat sprouts. However, we observed that NaCl treatment inhibited the progress and development of the sprouts, an issue we aim to address in future research. As shown in Figure 7, The proposed hypothetical model is illustrated in the schematic diagram below.

## Figures and Tables

**Table 1:** Primer sequences.

Primer	Sequences (5′-3′)	
*Actin*	F^1^: TCGTGAGAGATGACCCAGA	R^2^: CCGAGTCCAGCACAATACCT
*FePAL*	F: TCTCCAGAGCCGAAACAAG	R: AGCCTTGTTCCTGGATACAT
*FeC4H*	F: AACACACTACTCTCAGTTGC	R: ATTGGGTGATCGAGACTCTT
*Fe4CL*	F: CTCTTTCACGTCCACGGTTT	R: GATGATTTGGTGGATGGTGG
*FeCHS*	F: CGTCAAGCGTTTCATGATGT	R: CAAGGCTTGTGTTGACATGG
*FeCHI*	F: ACTTTGGAATCCGCTGTGAC	R: AGGCTTCAACATGGTGATCTG
*FeF3H*	F: CAAGGCTTGTGTTGACATGG	R: GACAGTGATCCAGGTCTTGC
*FeCAT*	F: GAGTTTGGTTCCCTTGCTT	R: TTCATACACTTCACTGGCGT
*FeSOD*	F: ATGGTGCTCCTGACGATG	R: CCACTGCCCTTCCAATAAT
*FePOD*	F: GTTCTGGTTGGGCTTGG	R: TTGTCCTCGTCTGTTGGTC
*FeSOS1*	F: CCTTACACCGTACCCGCTC	R: CCGGAAGAAACACAGCCAAC
*FeNHX1*	F: CGTTGCTAGGACGCAATGTT	R: ACAGTCCACGTCGGATGCC

F^1^: Forward primer classification; R^2^: Reverse primer categorization.

## Data Availability

The original contributions presented in this study are included in the article/supplementary material. Further inquiries can be directed to the corresponding author.

## Supplementary Materials

The following supporting information can be downloaded at: https://www.mdpi.com/article/10.3390/plants15060904/s1, Figure S1: Effects of different concentrations of NaCl treatment on buckwheat sprouts.

## References

[1] Mierziak J., Kostyn K., Kulma A. (2014). Flavonoids as Important Molecules of Plant Interactions with the Environment. Molecules.

[2] Ma M., Wang P., Yang R., Gu Z. (2018). Effects of UV-B Radiation on the Isoflavone Accumulation and Physiological-Biochemical Changes of Soybean During Germination: Physiological-Biochemical Change of Germinated Soybean Induced by UV-B. Food Chem..

[3] Chavarín-Martínez C.D., Reyes-Moreno C., Milán-Carrillo J., Perales-Sánchez J.X.K., Canizalez-Román V.A., Cuevas-Rodríguez E.O., López-Valenzuela J.A., Gutiérrez-Dorado R. (2022). Effect of Germination and UV-B Elicitation on Chemical Compositions, Antioxidant Activities, and Phytochemical Contents of Underutilised Mexican Blue Maize Seeds. Int. Food Res. J..

[4] Zhang J., Yang J., Yin Y. (2024). Germination Promotes Flavonoid Accumulation of Finger Millet (*Eleusine coracana* L.): Response Surface Optimization and Investigation of Accumulation Mechanism. Plants.

[5] Kim S.-H., Cui C.-B., Kang I.-J., Kim S.Y., Ham S.-S. (2007). Cytotoxic Effect of Buckwheat (*Fagopyrum esculentum* Moench) Hull Against Cancer Cells. J. Med. Food.

[6] Zheng R.-L., Wang J., Liu S.-Y., Sun Z.-P., Zhao L.-Y., Chen G.-T. (2024). Screening and Extraction Process Optimization For Potential A-Glucosidase Inhibitors From Quinoa Seeds. FMH.

[7] Xu L., Zhang L., Zhang S., Yang J., Zhu A., Sun J., Kalvakolanu D.V., Cong X., Zhang J., Tang J. (2024). Taxifolin Inhibits Melanoma Proliferation/Migration Impeding USP18/Rac1/JNK/β-catenin Oncogenic Signaling. Phytomedicine.

[8] Naponelli V., Piscazzi A., Mangieri D. (2025). Cellular and Molecular Mechanisms Modulated by Genistein in Cancer. Int. J. Mol. Sci..

[9] Liu Y.-F., Ling N., Zhang B., Chen C., Mo X.-N., Cai J.-Y., Tan X.-D., Yu Q.-M. (2024). Flavonoid-Rich Mulberry Leaf Extract Modulate Lipid Metabolism, Antioxidant Capacity, and Gut Microbiota In High-Fat Diet-Induced Obesity: Potential Roles of *FGF21* and *SOCS2*. FMH.

[10] Hu Y., Hou Z., Yi R., Wang Z., Sun P., Li G., Zhao X., Wang Q. (2017). Tartary Buckwheat Flavonoids Ameliorate High Fructose-Induced Insulin Resistance and Oxidative Stress Associated with The Insulin Signaling and Nrf2/HO-1 Pathways in Mice. Food Funct..

[11] Arus V.A., Georgescu A.M., Platon N., Rosu A.M., Muntianu G., Teusdea A.C., Vartolomei N., Nistor I.D. (2024). Preliminary Studies Concerning The Influence of Buckwheat Flour on The Quality of White Wheat Bread. Sci. Study Res. Chem. Chem. Eng..

[12] Ahmed A., Khalid N., Ahmad A., Abbasi N.A., Latif M.S.Z., Randhawa M.A. (2014). Phytochemicals and Biofunctional Properties of Buckwheat: A Review. J. Ind. Stud..

[13] Hu M., Yang J., Zhang J., Fang W., Yin Y. (2024). Physiology and Metabolism Alterations in Flavonoid Accumulation During Buckwheat (*Fagopyrum esculentum* Moench.) Sprouting. Plants.

[14] Dong N.-Q., Lin H.-X. (2021). Contribution of Phenylpropanoid Metabolism to Plant Development and Plant–Environment Interactions. J. Integr. Plant Biol..

[15] Zhuang W.-B., Li Y.-H., Shu X.-C., Pu Y.-T., Wang X.-J., Wang T., Wang Z. (2023). The Classification, Molecular Structure and Biological Biosynthesis of Flavonoids, and Their Roles in Biotic and Abiotic Stresses. Molecules.

[16] Jiao C., Yang R., Zhou Y., Gu Z. (2016). Nitric Oxide Mediates Isoflavone Accumulation and the Antioxidant System Enhancement In Soybean Sprouts. Food Chem..

[17] Alhaithloul H.A.S., Galal F.H., Seufi A.M. (2021). Effect of Extreme Temperature Changes on Phenolic, Flavonoid Contents and Antioxidant Activity of Tomato Seedlings (*Solanum lycopersicum* L.). PeerJ.

[18] Huang X., Li W., Wang J., Li Q., Shen Y., Cheng Y., Li T., Wang T., Wang Y., Song L. (2024). Nacl Stress on Physio-Biochemical, Phenolics Synthesis and Antioxidant System of Pea (*Pisum sativum* L.) Sprouts. FST.

[19] Yin Y., Hu J., Yang Z., Fang W., Yang J. (2023). Effects of methyl jasmonate and NaCl Treatments on the Resveratrol Accumulation and Defensive Responses in Germinated Peanut (*Arachis hypogaea* L.). Plant Physiol. Biochem..

[20] Lim I., Kang M., Kim B.C., Ha J. (2022). Metabolomic and Transcriptomic Changes in Mungbean (*Vigna radiata* (L.) R. Wilczek) Sprouts Under Salinity Stress. Front. Plant Sci..

[21] Zhang J.-S., Wang Y.-Q., Song J.-N., Xu J.-P., Yang H.-B. (2020). Effect of Aspartic Acid on Physiological Characteristics and Gene Expression of Salt Exclusion in Tartary Buckwheat Under Salt Stress. J. Plant Biochem. Biotechnol..

[22] Nam T.G., Kim D.-O., Eom S.H. (2018). Effects of Light Sources on Major Flavonoids and Antioxidant Activity in Common Buckwheat Sprouts. Food Sci. Biotechnol..

[23] Hao J., Wu T., Li H., Wang W., Liu H. (2016). Dual Effects of Slightly Acidic Electrolyzed Water (SAEW) Treatment on the Accumulation of γ-aminobutyric Acid (GABA) and Rutin in Germinated Buckwheat. Food Chem..

[24] Yin Y., Tian X., He X., Yang J., Yang Z., Fang W. (2022). Exogenous Melatonin Stimulated Isoflavone Biosynthesis in NaCl-Stressed Germinating Soybean (*Glycine max* L.). Plant Physiol. Biochem..

[25] Lin L.-Y., Peng C.-C., Yang Y.-L., Peng R.Y. (2008). Optimization of Bioactive Compounds in Buckwheat Sprouts and Their Effect on Blood Cholesterol in Hamsters. J. Agric. Food Chem..

[26] Ren S.-C., Sun J.-T. (2014). Changes in Phenolic Content, Phenylalanine Ammonia-Lyase (PAL) Activity, and Antioxidant Capacity of Two Buckwheat Sprouts in Relation to Germination. J. Funct. Foods..

[27] Kim S.-J., Zaidul I.S.M., Suzuki T., Mukasa Y., Hashimoto N., Takigawa S., Noda T., Matsuura-Endo C., Yamauchi H. (2008). Comparison of Phenolic Compositions Between Common and Tartary Buckwheat (*Fagopyrum*) Sprouts. Food Chem..

[28] Ling A., Li X., Hu X., Ma Z., Wu K., Zhang H., Hao M., Wei S. (2018). Dynamic Changes in Polyphenol Compounds, Antioxidant Activity, and PAL Gene Expression in Different Tissues of Buckwheat During Germination. J. Sci. Food Agric..

[29] Ma H., Xu X., Wang S., Wang J., Peng W. (2021). Effects of Microwave Irradiation on the Expression of Key Flavonoid Biosynthetic Enzyme Genes and the Accumulation of Flavonoid Products in *Fagopyrum tataricum* Sprouts. JCS.

[30] Seo J.-M., Arasu M.V., Kim Y.-B., Park S.U., Kim S.-J. (2015). Phenylalanine and LED Lights Enhance Phenolic Compound Production in Tartary Buckwheat Sprouts. Food Chem..

[31] Rai G.K., Mishra S., Chouhan R., Mushtaq M., Chowdhary A.A., Rai P.K., Kumar R.R., Kumar P., Perez-Alfocea F., Colla G. (2023). Plant Salinity Stress, Sensing, and Its Mitigation Through WRKY. Front. Plant Sci..

[32] Ahammed G.J., Li Z., Chen J., Dong Y., Qu K., Guo T., Wang F., Liu A., Chen S., Li X. (2024). Reactive Oxygen Species Signaling in Melatonin-Mediated Plant Stress Response. Plant Physiol. Biochem..

[33] Yin Y., Tian X., Yang J., Yang Z., Tao J., Fang W. (2022). Melatonin Mediates Isoflavone Accumulation In Germinated Soybeans (*Glycine max* L.) Under Ultraviolet-B Stress. Plant Physiol. Biochem..

[34] Ma M., Wang P., Yang R., Zhou T., Gu Z. (2019). UV-B Mediates Isoflavone Accumulation and Oxidative-Antioxidant System Responses in Germinating Soybean. Food Chem..

[35] Wang S., Wang J., Guo Y. (2018). Microwave Irradiation Enhances the Germination Rate of Tartary Buckwheat and Content of Some Compounds in Its Sprouts. Pol. J. Food Nutr. Sci..

[36] Lan H., Wang C., Yang Z., Zhu J., Fang W., Yin Y. (2024). The Impact of Lighting Treatments on the Biosynthesis of Phenolic Acids in Black Wheat Seedlings. Foods.

[37] Mu T., Han R. (2024). Magnesium and Nitrogen Co-Doped Carbon Dots Alleviate UV-B Radiation Damage in Wheat Seedlings. Pak. J. Bot..

[38] Liu J., Wang Q., Karagić Đ., Liu X., Cui J., Gui J., Gu M., Gao W. (2016). Effects of Ultrasonication on Increased Germination and Improved Seedling Growth of Aged Grass Seeds of Tall Fescue and Russian Wildrye. Sci. Rep..

[39] Peng W., Wang N., Wang S., Wang J., Bian Z. (2023). Effects of Microwave and Exogenous L-Phenylalanine Treatment on Phenolic Constituents, Antioxidant Capacity and Enzyme Inhibitory Activity of Tartary Buckwheat Sprouts. Food Sci. Biotechnol..

[40] Bian Z.-X., Wang J.-F., Ma H., Wang S.-M., Luo L., Wang S.-M. (2020). Effect of Microwave Radiation on Antioxidant Capacities of Tartary Buckwheat Sprouts. J. Food Sci. Technol..

[41] Zhuang L., Xu K., Zhu Y., Wang F., Guo L. (2020). Calcium Affects Glucoraphanin Metabolism in Broccoli Sprouts Under ZnSO_4_ Stress. Food Chem..

[42] Xue J., Quan X., Yang J., Fang W., Yin Y. (2024). Study on the Mechanism of Flavonoid Enrichment in Black Soybean Sprouts by Abscisic Acid/Melatonin Under Slight Acid Treatment. Foods.

[43] Yin Y., Liu C., Yang Z., Fang W. (2023). Ethephon Promotes Isoflavone Accumulation in Germinating Soybeans by Its Acceleration of Isoflavone Biosynthetic Pathway. Plant Physiol. Biochem..

[44] Mencin M., Jamnik P., Mikulič Petkovšek M., Veberič R., Terpinc P. (2022). Enzymatic Treatments of Raw, Germinated and Fermented Spelt (*Triticum spelta* L.) Seeds Improve the Accessibility and Antioxidant Activity of Their Phenolics. LWT.

[45] Huang C., Quan X., Yin Y., Ding X., Yang Z., Zhu J., Fang W. (2024). Enrichment of Flavonoids in Short-Germinated Black Soybeans (*Glycine max* L.) Induced by Slight Acid Treatment. Foods.

[46] Zhang H., Gao P., Fang H., Zou M., Yin J., Zhong W., Luo Z., Hu C., He D., Wang X. (2023). High-oleic Rapeseed Oil Quality Indicators and Endogenous Antioxidant Substances Under Different Processing Methods. Food Chem X.

[47] Gao F., Zhao H.-X., Yao H.-P., Li C.-L., Chen H., Wang A.-H., Park S.-U., Wu Q. (2016). Identification, Isolation and Expression Analysis of Eight Stress-Related R2R3-MYB Genes in Tartary Buckwheat (*Fagopyrum tataricum*). Plant Cell Rep..

[48] Yang M., He G., Hou Q., Fan Y., Duan L., Li K., Wei X., Qiu Z., Chen E., He T. (2022). Systematic Analysis and Expression Profiles of TCP Gene Family in Tartary buckwheat (*Fagopyrum tataricum* (L.) Gaertn.) Revealed the Potential Function of *FtTCP15* and *FtTCP18* in Response to Abiotic Stress. BMC Genom..

