# Supplementary material for: Effects of NaCl Treatment on Flavonoid Biosynthesis and Antioxidant System During Buckwheat Germination
Source: Plants (Basel). 2026 Mar 14;15(6):904. doi: 10.3390/plants15060904 (PMC13030649; doi:10.3390/plants15060904)
Supplement: Supplementary file 1 [file plants-15-00904-s001.zip › plants-4124661-supplementary.pdf]

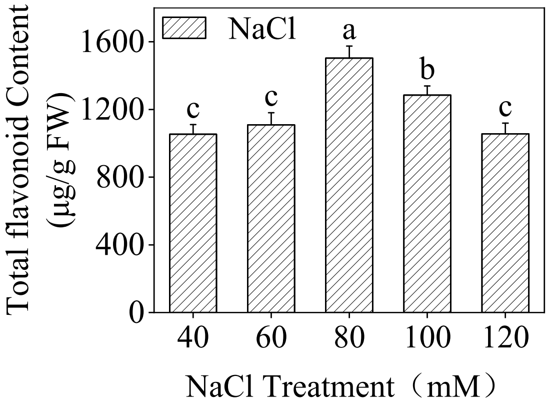

Figure S1. Effects of different concentrations NaCl treatment on buckwheat sprouts. Different lowercase letters within the same parameter indicate statistically significant differences among treatments within the same group. (one-way ANOVA with Tukey's test, $p < 0.05$).
